# Developmentally Regulated GTP binding protein 1 (DRG1) controls microtubule dynamics

**DOI:** 10.1038/s41598-017-10088-5

**Published:** 2017-08-30

**Authors:** Anna Katharina Schellhaus, Daniel Moreno-Andrés, Mayank Chugh, Hideki Yokoyama, Athina Moschopoulou, Suman De, Fulvia Bono, Katharina Hipp, Erik Schäffer, Wolfram Antonin

**Affiliations:** 1 Friedrich Miescher Laboratory of the Max Planck Society, Spemannstraße 39, 72076 Tübingen, Germany; 2 Institute of Biochemistry and Molecular Cell Biology, Medical School, RWTH Aachen University, 52074 Aachen, Germany; 3 Cellular Nanoscience, Center for Plant Molecular Biology (ZMBP), University of Tübingen, 72076 Tübingen, Germany; 4 Max Planck Institute for Developmental Biology, Spemannstraße 35, 72076 Tübingen, Germany

## Abstract

The mitotic spindle, essential for segregating the sister chromatids into the two evolving daughter cells, is composed of highly dynamic cytoskeletal filaments, the microtubules. The dynamics of microtubules are regulated by numerous microtubule associated proteins. We identify here Developmentally regulated GTP binding protein 1 (DRG1) as a microtubule binding protein with diverse microtubule-associated functions. *In vitro*, DRG1 can diffuse on microtubules, promote their polymerization, drive microtubule formation into bundles, and stabilize microtubules. HeLa cells with reduced DRG1 levels show delayed progression from prophase to anaphase because spindle formation is slowed down. To perform its microtubule-associated functions, DRG1, although being a GTPase, does not require GTP hydrolysis. However, all domains are required as truncated versions show none of the mentioned activities besides microtubule binding.

## Introduction

Microtubules are key cytoskeletal structures that play a vital role in a variety of cellular processes such as intracellular trafficking, regulation of cell polarity, cell shape maintenance, and chromatid segregation during cell division. Microtubules are polar assemblies built from α-/β-tubulin heterodimers, both of which are GTPases. The most prominent aspect of microtubules is their dynamic instability: Microtubules can shift rapidly between growth and shrinkage, especially at the plus tip. This instability is more pronounced during mitosis when the mitotic spindle forms.

Several types of microtubules are found in the mitotic spindle. The microtubules of the kinetochore, a protein complex assembled on centromeric chromatin, connect the centrosome with the kinetochore. Usually 20–30 kinetochore microtubules are bundled into stable k-fibers, which mediate chromosomal movement. The non-kinetochore microtubules are part of the spindle body, without being attached to the kinetochore. They are important for separating the poles and mitotic spindle stability. Lastly, astral microtubules radiate from the centrosomes toward the cell cortex and position the spindle (reviewed in detail in refs 1, 2).

In the presence of GTP, pure α/β tubulin dimers are sufficient to generate microtubules *in vitro*. In cells, however, nucleating factors are additionally required (reviewed in detail in refs 1–3). For the mitotic spindle, centrosomes are the most prominent nucleation centers but other nucleation pathways also exist. Microtubules can nucleate around chromosomes, a process which is regulated by the small GTPase Ran. Additionally, microtubules can nucleate from already existing microtubules within the spindle. These other pathways can predominate if no centrosomes are present, e.g. during the second meiotic division in vertebrates, or when centrosomes are artificially removed. However, these other pathways are also crucial for timely spindle assembly in the presence of centrosomes. The additional nucleation pathways increase the probability that a microtubule finds a kinetochore by elevating microtubule density around chromosomes. In addition, other microtubule associated proteins, and mechanical processes such as cell rounding, are also involved in spindle assembly and facilitate the microtubule-kinetochore attachment during mitosis^1, 2^.

Several classes of microtubule-associated proteins are known. These include microtubule polymerases and de-polymerases, nucleation factors, severing enzymes, microtubule bundling/crosslinking proteins, motor proteins that are essential to establish the bipolar array e.g. by sliding microtubules, microtubule capping/end-binding/tracking factors and many more (reviewed in ref. 3). The further elucidation of unknown pathways and factors involving microtubules is challenging due to a high degree of redundancy in mitotic spindle processes. However, errors in chromosome segregation occur more often even when the functions of single factors are removed. The presence of numerous diverse yet partially redundant factors and pathways most likely represents an inbuilt security mechanism of the cell. Hence, it is crucial as well as challenging to identify such partially redundant factors during spindle assembly and maintenance, which are deregulated in many disease contexts^4–6^.

Here, we identify Developmentally regulated GTP-binding protein 1 (DRG1) as a microtubule polymerase that also bundles and stabilizes microtubules. Developmentally regulated GTP-binding proteins (DRGs) are a deeply conserved group of proteins belonging to the subfamily of Obg GTPases^7^. They were independently identified in a variety of organisms in the 1990’s^8–14^. DRGs are conserved from archaebacterial having one DRG to eukaryotes from yeast to human, containing DRG1 and DRG2^15^. Plants even have three DRGs^16^. Beside the canonical G-domain they do not share similarities with other known GTPases and their function is still largely unclear. As DRG1 is highly upregulated in mouse embryonic brain it was suggested to act as a developmental factor^9^. However, DRGs are also expressed widely in adult tissue^8, 15, 17^.

DRGs associate with DRG family regulatory proteins (DFRPs) which stabilize DRGs and prevent their ubiquitination and degradation by the proteasomes^18, 19^. Consistently, DRG1 is substantially downregulated after DFRP1 knock-down. While DRG1 and DRG2 are highly similar (58% identity for human proteins), the two DFRPs, DFRP1 and DFRP2 share only similarities in their DFRP domain. This domain is important for DRG interaction but the binding area extends further^20^. DFRP1 binds specifically to DRG1 while it is under debate if DFRP2 binds exclusively to DRG2 or also to DRG1^18, 19, 21^. Like other Obg GTPases, DRG1 and its interaction partner DFRP1 might be involved in translation because they co-sediment with polysomes^19–22^ and bind RNAs^17^. However, the precise role of DRG1 in the process is ambiguous. The crystal structure of the yeast DRG1 homolog, Rbg1 (Ribosome binding GTPase 1), together with a C-terminal fragment of the yeast homolog of DFRP1 (Tma46), shows that the canonical G-domain of the DRGs is interrupted by another domain, the S5D2L domain^20^. DRG1 seems to have an intrinsic GTPase activity that does not necessarily need a GTPase activating protein as is usually the case for most small GTPases^16, 20, 23^. Potassium ions stimulate this activity as well as DFRP1 binding. It is unclear whether DFRP1 functions as a GTPase activating protein as it binds opposite to the GTP binding pocket suggesting it stimulates the GTPase activity differently e.g. by improving the affinity to potassium ions. Despite their deep conservation, the functions of DRGs are still mostly unknown, though previous studies have suggested roles in development and cell growth^9, 24, 25^.

Here, we identify Developmentally regulated GTP-binding protein 1 (DRG1) as a microtubule binding protein. Using *in vitro* approaches, we show that DRG1 bundles and stabilizes microtubules. Furthermore, DRG1 can promote polymerization of microtubules. Consistent with this observation, DRG1 is involved in spindle dynamics in human cells.

## Results

### DRG1 directly interacts with microtubules

**Figure 1:**
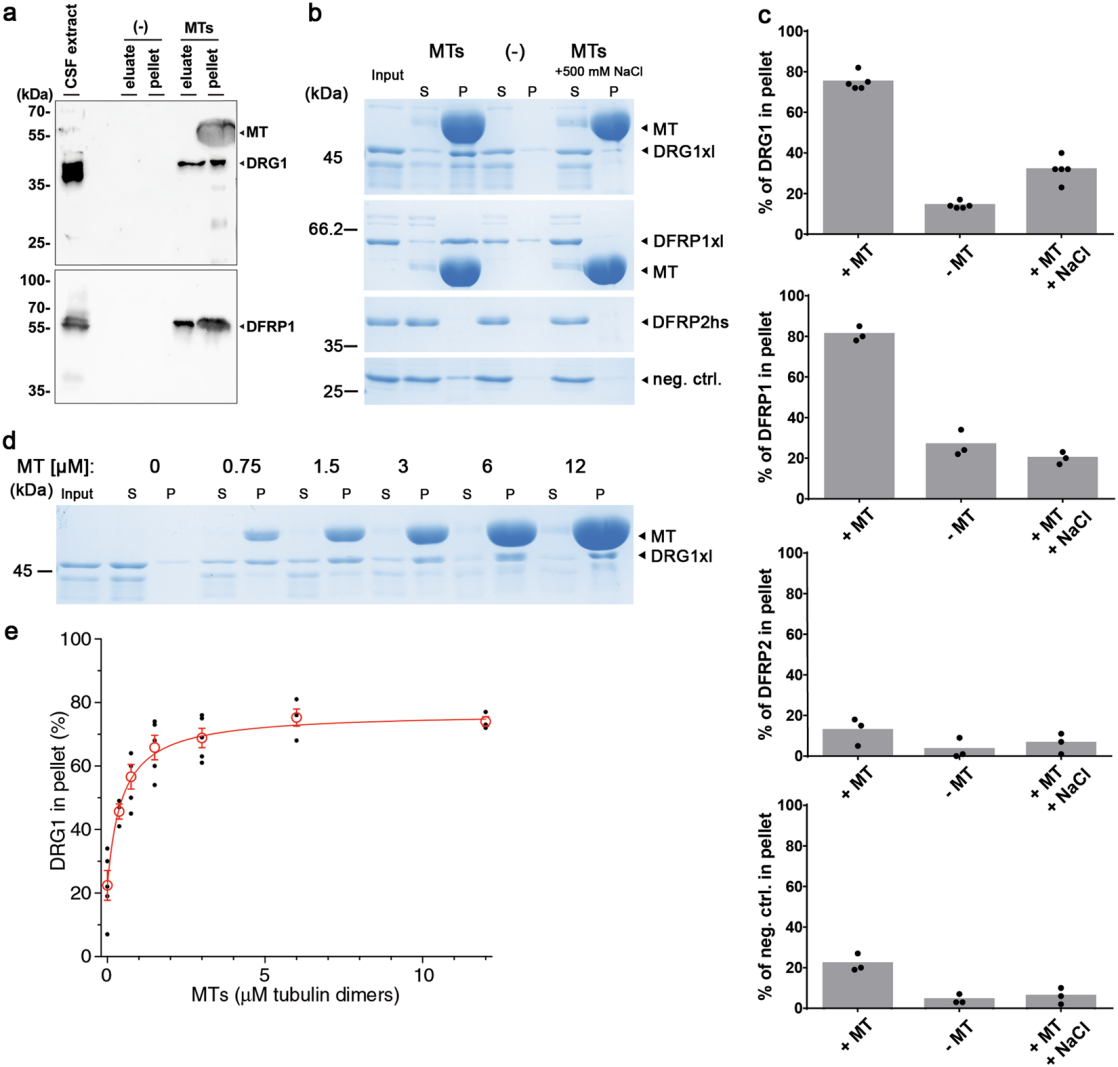
DRG1 and DFRP1 bind microtubules. **(a)** 4 µM taxol-stabilized microtubules (MTs) were incubated with *Xenopus* cytostatic factor arrested (CSF) extract. Microtubules were co-sedimented together with MT-binding proteins and eluted by 500 mM NaCl in CSF-XB buffer. The pellet and the elution were analyzed by western blotting. **(b)** Recombinant *Xenopus laevis* DRG1 and DRFP1 as well as human DFRP2 were incubated with 12 µM taxol-stabilized microtubules to test if the observed binding is direct. RanQ69L served as a negative control (neg. ctrl.). S: supernatant, P: pellet. **(c)** Coomassie stainings of recombinant proteins in binding experiments as in **(b)** were quantified using ImageJ. The columns represent the averages of the protein fractions found in the pellet from at least three different experiments with the individual data points indicated. **(d)** Recombinant *Xenopus laevis* DRG1 was incubated with different concentrations of taxol-stabilized microtubules. **(e)** Coomassie staining of recombinant DRG1 from **(d)** was quantified using ImageJ and blotted in dependence to the microtubule concentrations. The binding curve was fitted to the data points calculating a K_D_ of 0.47 (+/−0.05) µM.

DRG1 has been recently shown to localize at the mitotic spindle^25^, which raises the question whether the protein can interact with microtubules. To test this, *Xenopus laevis* egg extracts, arrested in a mitotic state, were incubated with polymerized, taxol-stabilized microtubules. After sedimentation of the microtubules by centrifugation, the tubulin-bound fraction was eluted with a high salt buffer (Fig. 1a). Whereas DRG1 and its interaction partner DFRP1 were not pelleted in the absence of microtubules, both proteins were found in the pellet fraction in the presence of microtubules. Both, DRG1 and DFRP1 were eluted with high salt from microtubules indicating that they bind specifically to microtubules. Similar results were obtained from experiments using HeLa nuclear extracts (Supplementary Fig. S1a, only the eluate is shown). DRG1 and DFRP1 can be pelleted with microtubules and eluted with high salt, similar to two known microtubule-associated proteins MEL28/ELYS and chTOG, the human homolog of XMAP215. In contrast, we did not find the chromatin-associated condensin subunit CAP-G and DFRP2 in the microtubule-bound fraction.

To test whether DRG1 and DRFP1 bind directly to microtubules we incubated taxol-stabilized microtubules with recombinant DRG1, DRFP1 or DFRP2 (Fig. 1b and c, purified proteins are shown in Supplementary Fig. S1b). Whereas DRG1 and DFRP1 pelleted with microtubules, DFRP2 and a negative control protein remained in the supernatant. Addition of 500 mM NaCl to the incubation buffer prevented DRG1 and DFRP1 microtubule association indicating that the binding is specific and occurs via polar/charge interactions. Titration of the microtubule amount in the microtubule pelleting assay showed that DRG1 can be saturated and has a dissociation constant K_D_ of 0.47 (+/−0.05) µM at 1 µM DRG (Fig. 1d and e).

### DRG1 diffuses on microtubules

**Figure 2:**
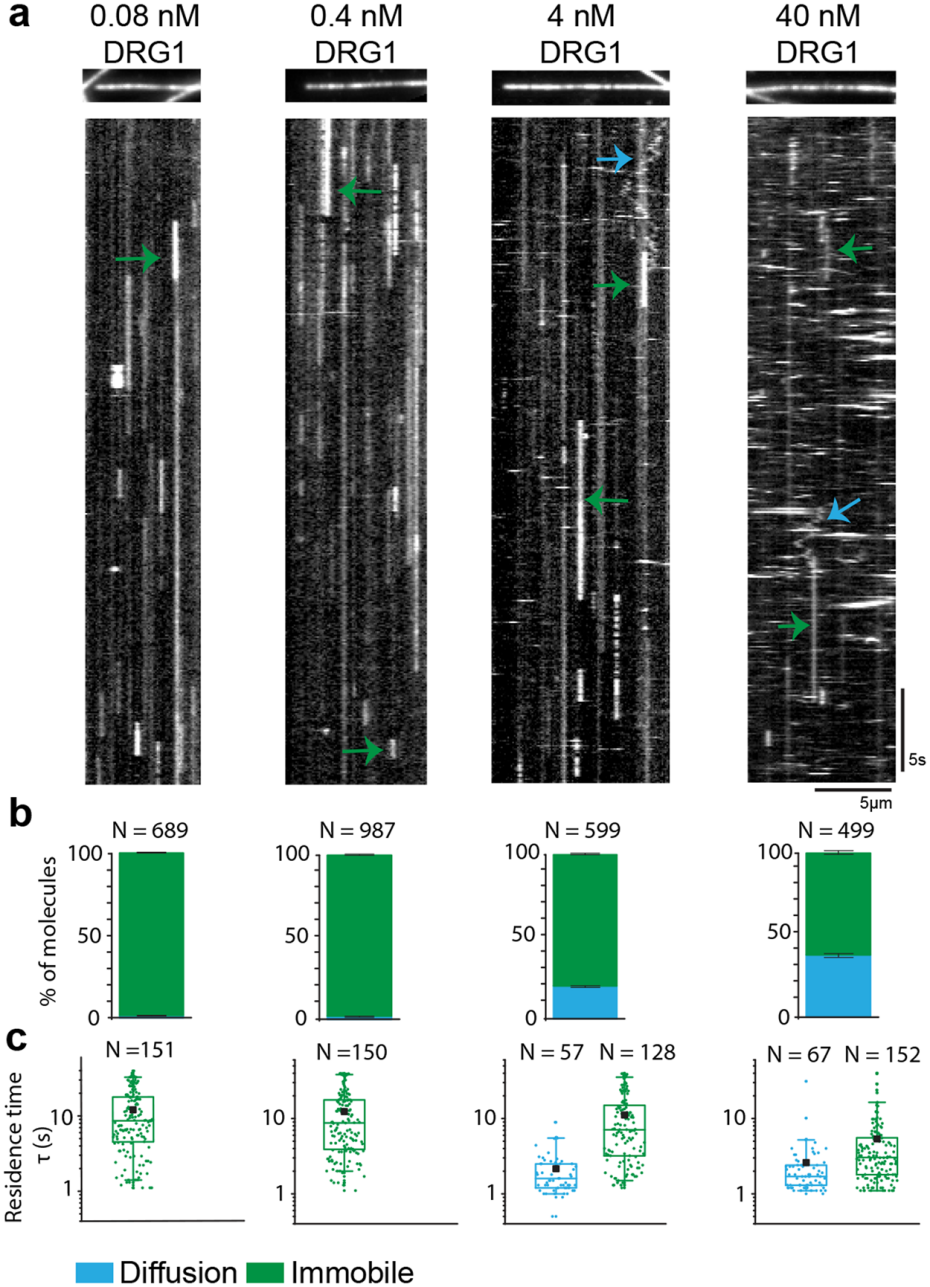
DRG1 interacts with the microtubule lattice in distinct binding modes. **(a)** Kymographs showing two different binding modes (diffusion and immobile) of eGFP-DRG1 over four different concentrations (0.08 nM, 0.4 nM, 4 nM, 40 nM). On top of each kymograph, the respective image of the rhodamine-labelled microtubule is shown. Every kymograph represents a microtubule on its horizontal axis observed over time (vertical). **(b)** The proportions of the different DRG1 binding populations are shown at the aforementioned concentrations. **(c)** Residence times of diffusive and immobile DRG1 molecules on microtubule lattice are 12.0 ± 0.8 s (mean ± S.E.M., 0.08 nM), 12.4 ± 0.9 s (0.4 nM), 11.1 ± 0.9 s (4 nM), 5.4 ± 0.6 s (40 nM) for the immobile fraction and 2.2 ± 0.2 s (4 nM) and 2.6 ± 0.5 s (40 nM) for the diffusive population. Color scheme: diffusion (cyan), immobile (green). Exemplary events are pointed out by arrows.

To confirm and characterize DRG1 binding to microtubules further, we used a total-internal-reflection-fluorescence (TIRF) microscopy-based assay to observe the DRG1 binding and mobility with single-molecule resolution. We observed that DRG1 interacted with microtubules in two different ways (see methods and Fig. 2a): DRG1 transiently bound to microtubules either in an immobile (green arrows) or diffusive manner (cyan arrows). Note that fluorescent signals that appear as small horizontal stripes in the kymographs of Fig. 2a are due to non-specific, transient encounters of larger molecules that diffuse in 3D and come into proximity of the surface (Supplementary Fig. S2). Such events typically lasted only for one frame with an image acquisition time of 0.1 s. For the specific interactions, we analyzed the relative proportions of DRG1 binding modes as a function of the DRG1 concentration (Fig. 2b). With increasing DRG1 concentrations from 80 pM to 40 nM, we observed an increase of the DRG1 fraction showing diffusive microtubule binding and, conversely, a decrease in the proportion showing immobile binding. We calculated the average residence or dwell time – i.e. the average time that a DRG1 molecule spends on the microtubule lattice – for the different populations. Note that the inverse of the average residence time is the off-rate constant. For the immobile DRG1 species, the average residence time decreased from about 12 s to 5 s, while the residence time of the diffusive DRG1 population increased slightly with increasing DRG1 concentrations. Interestingly, different DRG1 intensities visible on the kymograph suggest that DRG1 may bind microtubules not only as a monomer but also as a multimer. However, our signal-noise-ratio did not allow us to quantify oligomerization based on the fluorescence emission.

To test if the two binding modes represent different nucleotide binding states of DRG1, we repeated the experiment in the presence of the non-hydrolysable GTP analogue GTPγS (Supplementary Fig. S3). DRG1 also bound to microtubules in the presence of GTPγS. We did not see a significant difference in the proportions of the diffusive versus the immobile DRG1 populations in comparison to experiments performed in the presence of GTP suggesting that DRG1 binding to microtubules is not determined by the nucleotide state of DRG1. For the lower concentrations of DRG1, the residence times of the immobile population of DRG1 decreased slightly in the presence of GTPγS compared to GTP. The diffusive movements on the microtubule lattice has also been observed for the plus-end tracking protein EB1^26^ or for the MCAK motor protein, a microtubule depolymerase that diffuses on the microtubule to target both ends and performs its function there^27^. In both cases, the diffusion facilitates “end-finding” of the microtubules and thus, increases the concentration of the proteins at the microtubule ends as compared to random diffusion in solution. We did not observe any preference for end binding on taxol-stabilized microtubules (Fig. 2a). However, these microtubules do not have a GTP cap at the end. To mimic the GTP cap, we used microtubules grown in the presence of GTPγS. However, also for these microtubules we did not observe a qualitative difference in the binding behavior (Supplementary Fig. S4). Since microtubule binding did not depend on the nucleotide state of DRG1 or the microtubules, the different behavior might be due to oligomerization of DRG1 or due to the interaction of different binding domains.

### DRG1 binds microtubules via multiple regions

**Figure 3:**
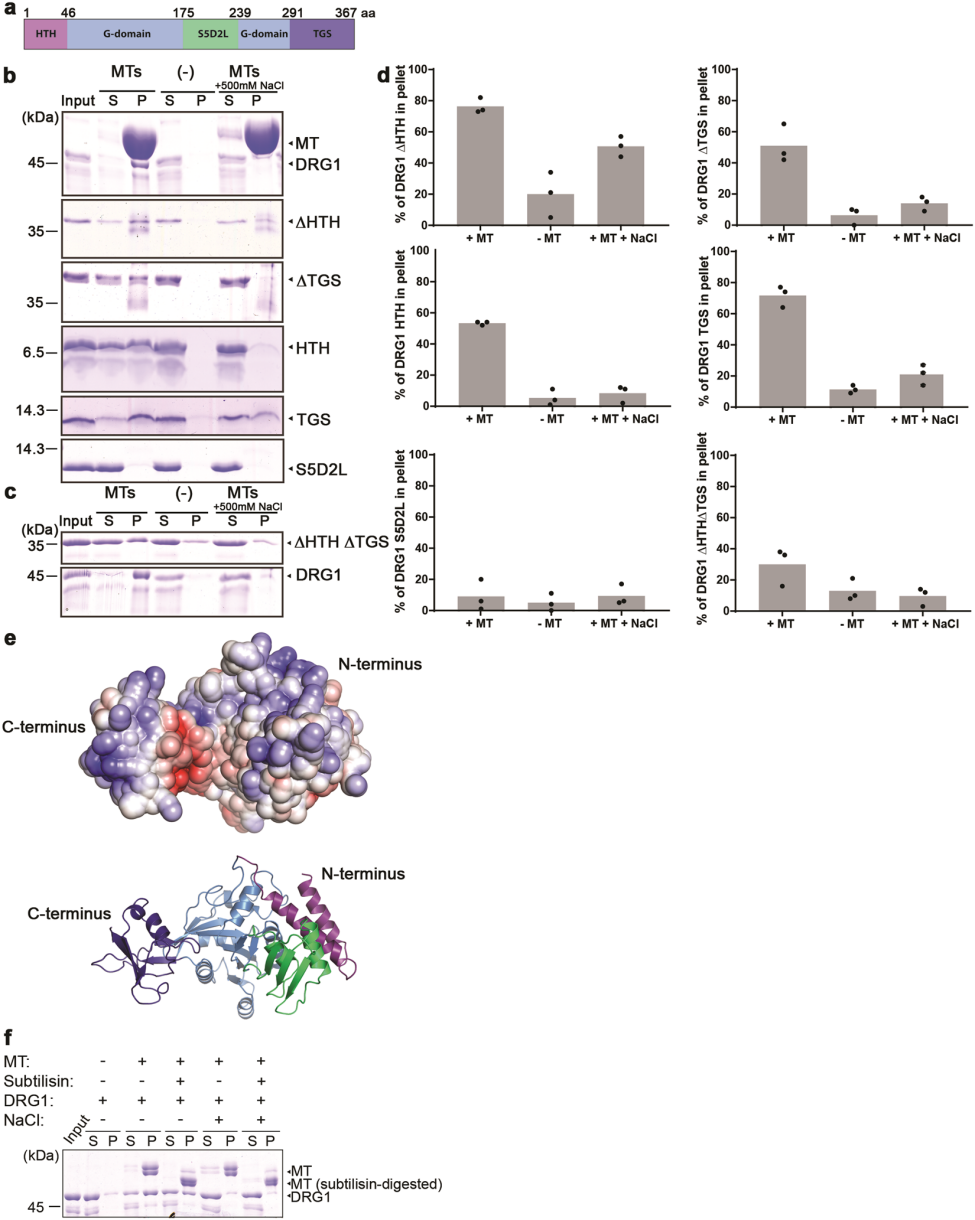
Different DRG1 domains interact with microtubules. **(a)** Scheme of DRG1 indicating the different domains. **(b)** Full-length and truncated versions of *Xenopus laevis* DRG1 were incubated and co-sedimented with taxol-stabilized MTs as in Fig. 1b. **(c)** Full-length DRG1 and its truncated versions lacking both the HTH and TGS domains were incubated and co-sedimented with taxol-stabilized MTs. **(d)** Coomassie stainings of recombinant proteins in binding experiments as in **(b)** and **(c)** were quantified using ImageJ. The column represents the average of the protein fractions found in the pellet from three different experiments with the individual data points indicated **(e)** Structure prediction of *Xenopus* DRG1 modeled with Swiss-Model^46^. Blue color represents the positively charged surface and red the negative charges (±5 kT/e). Lower structure shows a cartoon representing the different domains using the color code from **(a)**. **(f)** Taxol-stabilized MTs were digested by the protease subtilisin and employed in the co-sedimentation assay with full-length DRG1.

Having observed a direct microtubule interaction of DRG1, we were wondering which domains of the protein are required for microtubule binding. DRG1 consists of an N-terminal helix-turn-helix (HTH) motif, followed by the GTPase domain, which is interrupted by the S5D2L domain; the TGS domain constitutes the C-terminal part of the protein^20^ (Fig. 3a). As observed in Fig. 1, full-length DRG1 pelleted together with taxol-stabilized microtubules in a high salt sensitive manner (Fig. 3b). The truncated proteins lacking the N-terminal HTH or the C-terminal TGS domain were similarly pelleted with microtubules. A varying fraction, depending on the truncation, was also in the supernatant indicating a weaker and differential association with microtubules (Fig. 3d). Interestingly, both the HTH and the TGS domain individually bound microtubules whereas the isolated S5D2L domain did not show this association. We also detected salt sensitive microtubule binding for a truncated DRG1 version lacking both the HTH and TGS domain, indicating that the GTPase domain of DRG1 also interacts with microtubules (Fig. 3c). These results show that several domains of DRG1 are able to bind microtubules. We modeled the *Xenopus* DRG1 structure based on the available yeast Rbg1 structure (Fig. 3e). When calculating the electrostatic surface potential, we found an extensive positively charged surface formed by parts of the TGS, the HTH, the S5D2L and the G-domain opposite of the GTP-binding site as previously observed for Rbg1^20^. As microtubule-associated proteins often interact with microtubules via positively charged domains this entire region might be the microtubule binding site of DRG1.

### DRG1 binds to microtubules lacking the negatively charged C-terminus of tubulin

Many microtubule-binding proteins bind tubulin via its acidic, negatively charged, unstructured C-terminus, which is also the site of many posttranslational modifications^28, 29^. This C-terminus can be cleaved off by the protease subtilisin. Repeating the microtubule co-sedimentation assay using subtilisin-digested microtubules showed that DRG1 still bound tubulin lacking the negatively charged C-terminus although the binding affinity might have been reduced (Fig. 3f).

### DRG1 bundles microtubules

**Figure 4:**
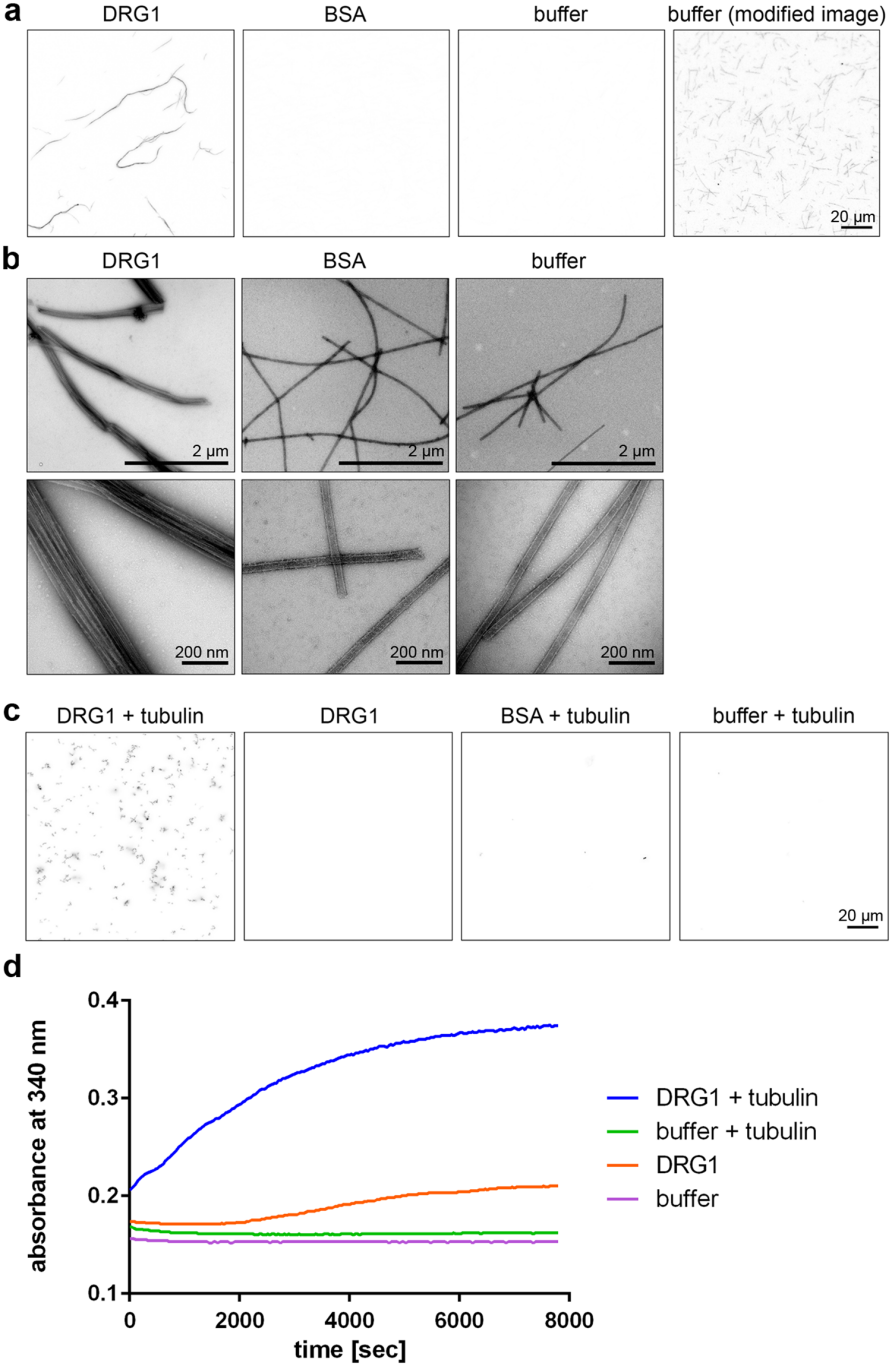
DRG1 bundles and polymerizes microtubules *in vitro* **(a,b)** 0.3 µM taxol-stabilized, Cy-3 labeled MTs were incubated with 1 µM DRG1, BSA or buffer for 10 min at RT. Samples were analyzed by confocal microscopy **(a)** or electron microscopy **(b)**. **(c)** 5 µM tubulin (mixed in a 1:4 Cy3 labeled:unlabeled ratio) were incubated with 1 mM GTP and 1 µM DRG1, BSA or buffer for 30 min at 37 °C, fixed with BRB80 buffer containing 0.25% glutaraldehyde, 10% glycerol and 0.1% Triton X-100, spun down on coverslips and post-fixed with cold methanol. Samples were analyzed by confocal microscopy. MT only polymerized if DRG1 was present. **(d)** Light-scattering experiments were carried out by mixing 2.5 µM tubulin, 1 mM GTP and 1 µM DRG1 in a 96-well plate which was followed by immediately measuring absorbance at 340 nm every 38 seconds for 2:10 hours.

Since multiple domains of DRG1 were binding microtubules, we tested whether DRG1 could bundle them. To this end, we incubated taxol-stabilized, fluorescently-labeled microtubules with DRG1. Addition of 1 µM recombinant DRG1 induced microtubule bundling as observed by fluorescence microscopy (Fig. 4a). Electron microscopy analysis confirmed microtubule bundling in the presence of DRG1 (Fig. 4b). This bundling activity is consistent with DRG1 having multiple microtubule binding sites.

### DRG1 promotes microtubule polymerization

Many microtubule binding proteins regulate microtubule dynamics^3^. When we added DRG1 to a fluorescently-labeled tubulin solution provided below the critical concentration for spontaneous microtubule growth^30^, we observed microtubule polymerization. A control without DRG1 showed no microtubule growth (Fig. 4c). We confirmed this observation by light scattering experiments: polymerization of tubulin at a relatively low concentration of 2.5 µM was observed when DRG1 was added (Fig. 4d). Thus, DRG1 is a GTPase that induces microtubule polymerization.

### DRG1 stabilizes microtubules

**Figure 5:**
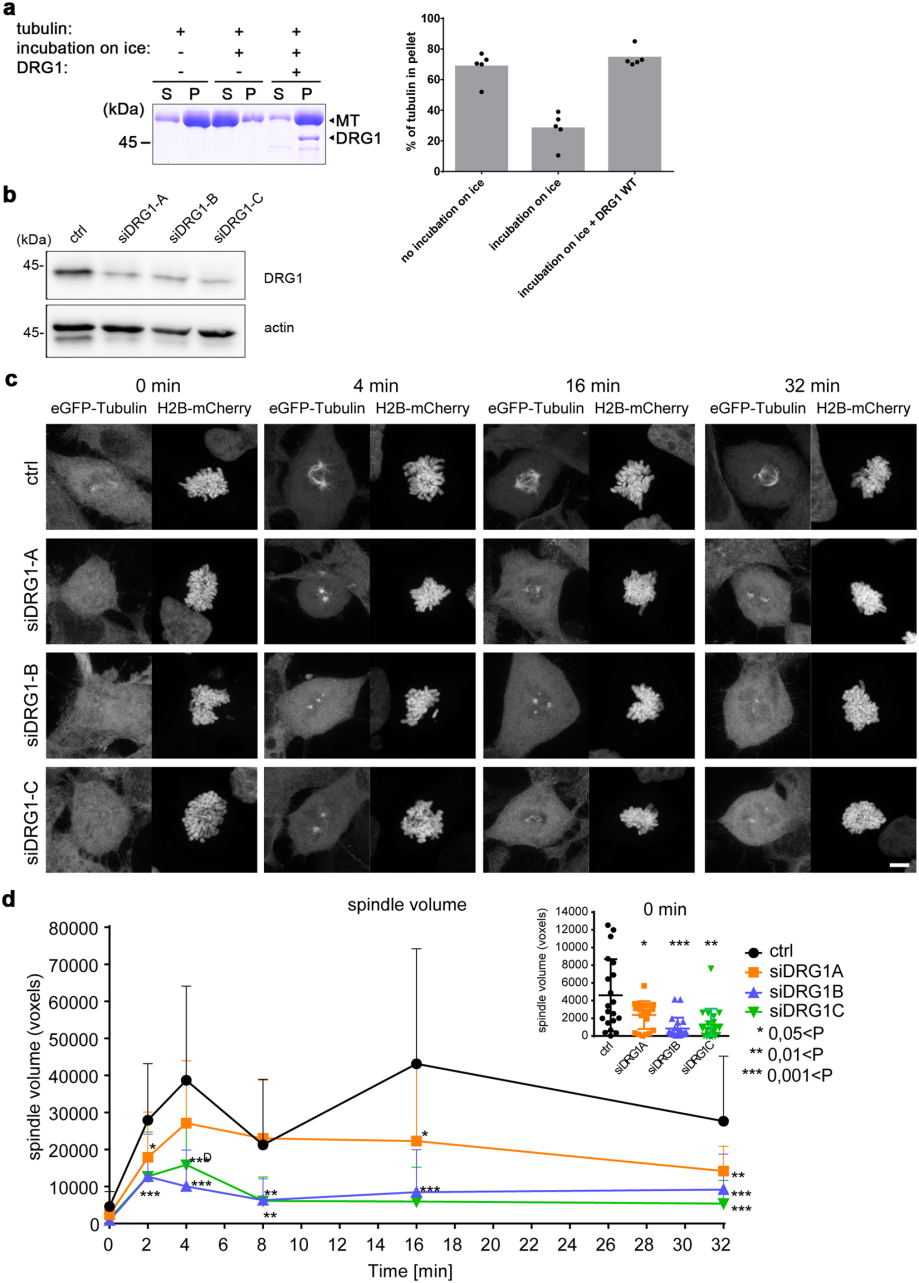
DRG1 stabilizes microtubules in the cold. **(a)** 12 µM tubulin was polymerized in the absence or presence of 5 µM DRG1 for 1 h at 37 °C and placed on ice for 30 min. MTs were then pelleted by centrifugation, while free tubulin stays in the supernatant. Pellet and supernatant were analyzed by SDS-PAGE. The quantification shows the tubulin fraction found in the pellet. Columns represent the average of five independent experiments with the individual data points indicated. **(b)** Western blotting shows that DRG1 was knocked-down in HeLa cells stably expressing histone H2B-mCherry and eGFP-tubulin by siRNA 72 h post-transfection. **(c)** siRNA treated HeLa cells were placed 72 hrs after transfection on ice for 1 hour to induce MT disassembly. Warm medium was then added to the cells which were fixed with 4% PFA at indicated time points. Maximum intensity projections of Z-stacks from representative prometaphase cells at the given time points are shown. **(d)** The spindle size in voxels was quantified in 20 random prometaphase cells (from 2 independent experiments) per time point and siRNA (mean and SD are plotted). ***P < 0.001; **P < 0.01; *P < 0.01. Insert shows the spindle size at 0 min enlarged.

The bundling and polymerization activities of DRG1 could indicate that DRG1 might also have a stabilizing effect on microtubules. To test whether DRG1 stabilizes microtubules, we polymerized microtubules from a high concentration of tubulin (12 µM) in the presence or absence of DRG1 at 37 °C for one hour and afterwards placed the sample on ice for 30 minutes. Note that we did not use taxol. Polymerized and stabilized microtubules were pelleted by centrifugation. The microtubules polymerized efficiently under these conditions but disassembled upon placing on ice in the buffer control. In the presence of DRG1, microtubules remained in the polymerized state despite the incubation on ice (Fig. 5a). The effect was dependent on the DRG1 concentration showing full microtubule stabilization at 5 µM DRG1 (Supplementary Fig. S5).

This *in vitro* stabilization effect was confirmed in HeLa cells stably expressing histone H2B-mCherry and eGFP-tubulin. For this purpose, DRG1 expression was downregulated by siRNA for 72 hrs (Fig. 5b). Afterwards, the cells were placed for one hour on ice which induced spindle disassembly in the mitotic population. Then, warm medium was added and the re-growth of microtubules was analyzed by fixing and analyzing the samples at different time points. Microtubules re-grew much slower in cells with reduced levels of DRG1 compared to the control cells (Fig. 5c and d). Thus, mitotic spindles recover much faster after a cold shock in cells having endogenous DRG1 levels suggesting that DRG1 either accelerates microtubule re-polymerization once warm medium is added, or DRG1 prevents the complete disassembly of the spindle upon cold treatment. Noticeably, remnants of the mitotic spindle are often observed in control cells after 1 h on ice (insert Fig. 5d, 0 min) but less frequent in cells lacking DRG1. These remnants might cause a faster re-assembly of the mitotic spindle. Both hypotheses are in agreement with our *in vitro* findings that DRG1 promotes microtubule polymerization and stabilization.

### The GTPase activity of DRG1 is not necessary for its microtubule functions

**Figure 6:**
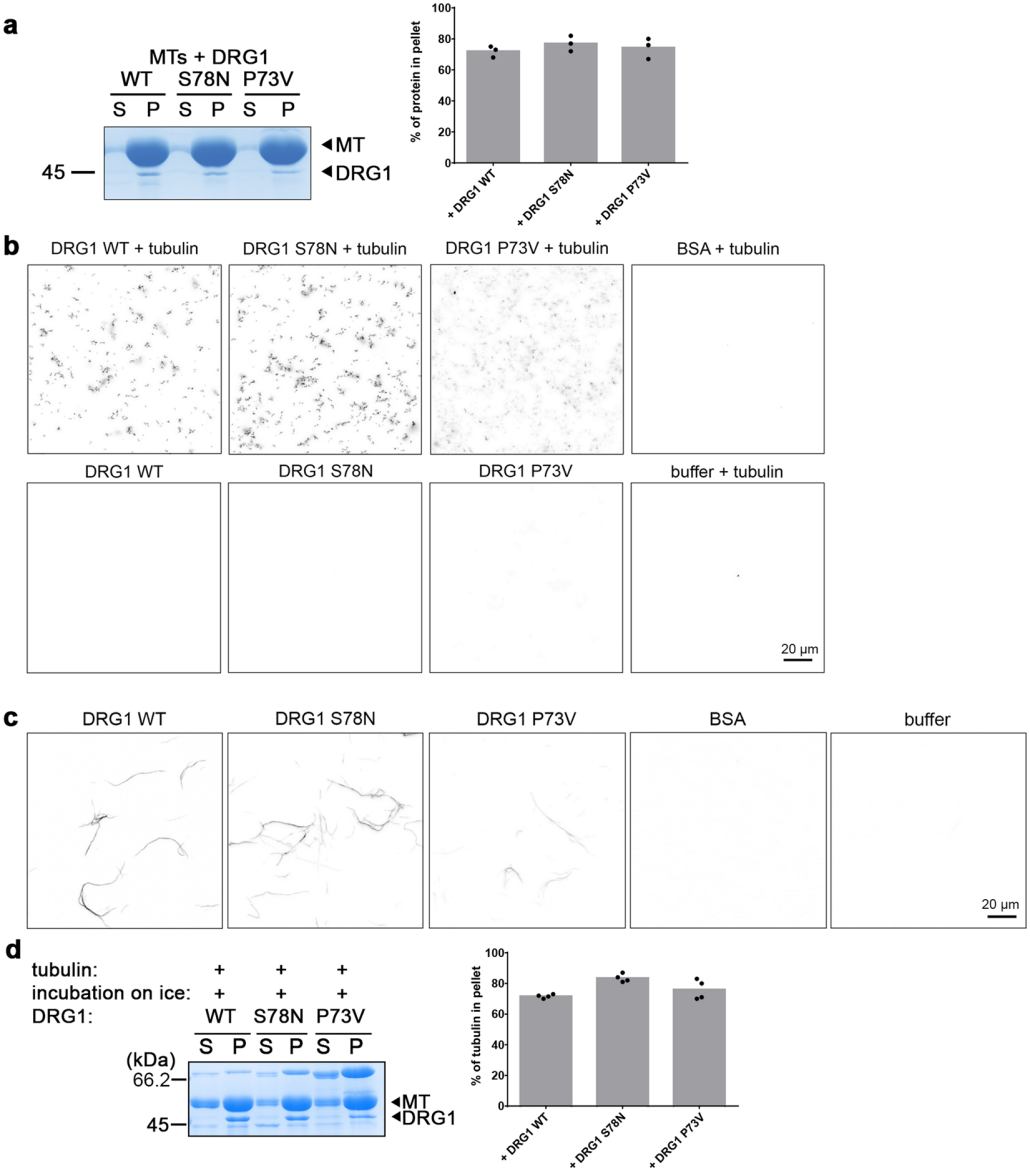
Microtubule binding, bundling, polymerization and stabilization activity of DRG1 does not require GTP hydrolysis. **(a)** MT co-sedimentation with 12 µM taxol-stabilized microtubules and DRG1 WT, DRG1 S78N and DRG1 P73V. The quantification shows the fraction of the proteins in the pellet. Columns represent the average of three independent experiments, individual data points are indicated. **(b)** MT-polymerization assay was done as in Fig. 4 using 1 µM DRG1 WT, S78N and P73V. **(c)** MT bundling assay was done as in Fig. 4 using 1 µM DRG1 WT, S78N and P73V. **(d)** DRG1 S78N and DRG1 P73V were employed in the microtubule stabilization assay as in Fig. 5. The quantification shows the tubulin fraction found in the pellet. Columns represent the average of four independent experiments, individual data points are indicated.

DRG1 is a member of the small GTPase superfamily. To test whether its GTP binding and hydrolyzing activity is required for its microtubule functions, we used a dominant positive *Xenopus* DRG1 mutant, with a P73V exchange in the G1 box of the GTPase domain, which stabilizes the GTP-bound state^31^, and a dominant negative mutant, DRG1 S78N, which represents the GDP- bound or nucleotide free state of the GTPase^20, 22^. Both mutants were still able to bind microtubules (Fig. 6a, Supplementary Fig. S6a), which is consistent with our observation that several domains are able to bind microtubules on their own (Fig. 3b and d). This indicates that the GTPase activity of DRG1 is not required for microtubule binding. Indeed, no difference was observed when GTP was replaced in the microtubule pelleting assay by the non-hydrolysable analogue GTPγS (Supplementary Fig. S6b). This is consistent with the TIRF based assays which showed not significant difference in DRG1 microtubule binding and mobility between GTP and GTPγS (Fig. 2 and Supplementary Fig. S3).

The DRG1 S78N and P73V mutants are also able to polymerize tubulin (Fig. 6b, Supplementary Fig. S6c) and bundle (Fig. 6c) as well as to stabilize microtubules (Fig. 6d). This suggests that DRG1 does not require its GTPase activity for its microtubule-associated functions.

### Full-length DRG1 is necessary to bundle, polymerize and stabilize microtubules

As shown above, most truncated versions of DRG1 were capable of binding microtubules. We were curious to see if they are also able to bundle, polymerize and stabilize microtubules. Therefore, we repeated the previously described assays using the recombinant DRG1 fragments. Fragments lacking the HTH, the TGS domain or both, as well as the HTH, the TGS or the S5D2L domain individually were neither able to bundle microtubules (Supplementary Fig. S7a), nor promoted polymerization (Supplementary Fig. S7b) nor stabilized them upon cold stress (Supplementary Fig. S7c) under the same conditions used for the wild-type (Figs 4 and 5a). It was observed before in a different context that the full-length protein is necessary for its *in vivo* function^20^.

### DRG1 impacts spindle dynamics in cells

**Figure 7:**
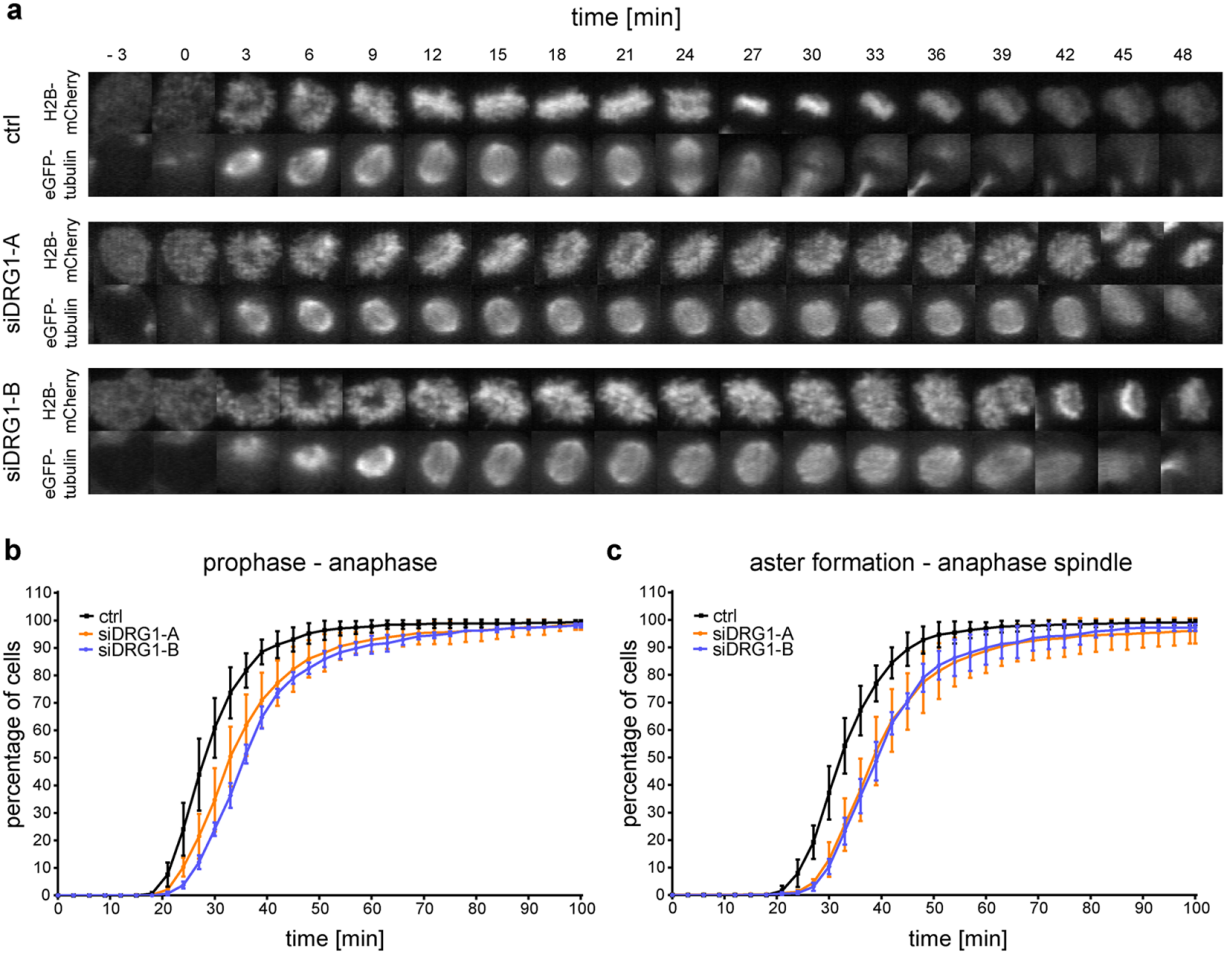
DRG1 regulates mitotic progression and spindle assembly in cells. **(a)** HeLa cells stably expressing histone H2B-mCherry and tubulin-eGFP were transfected with siRNA oligonucleotides against DRG1 or a control. The cells were imaged every 3 min for 48 h starting at 24 h post-transfection. **(b)** A cumulative histogram of the timing from prophase to anaphase onset based on chromatin morphology (based on H2B-mCherry) and **(c)** of the timing from aster formation to anaphase spindle (based on eGFP-tubulin) are shown. Mean and SD from 3 independent experiments containing more than 150 cell trajectories per siRNA and experiment are plotted.

Our results show that DRG1 influences microtubule behavior. To assess its impact on microtubule dynamics in cells, we analyzed HeLa cells stably expressing histone H2B-mCherry and tubulin-eGFP while passing through mitosis. DRG1 expression was down-regulated by siRNA. 24 hours post-transfection, live-cell imaging was carried out for 48 hours (Fig. 7a). Analysis of chromatin features using the software CellCognition^32^ showed that the time from prophase to anaphase onset was extended upon DRG1 downregulation as compared to the control conditions (Fig. 7b). Analyzing the spindle features showed that a partial knock-down of DRG1 does not change the size or intensity of the spindle (data not shown), but the time from aster to the anaphase spindle formation was extended (Fig. 7c).

**Figure 8:**
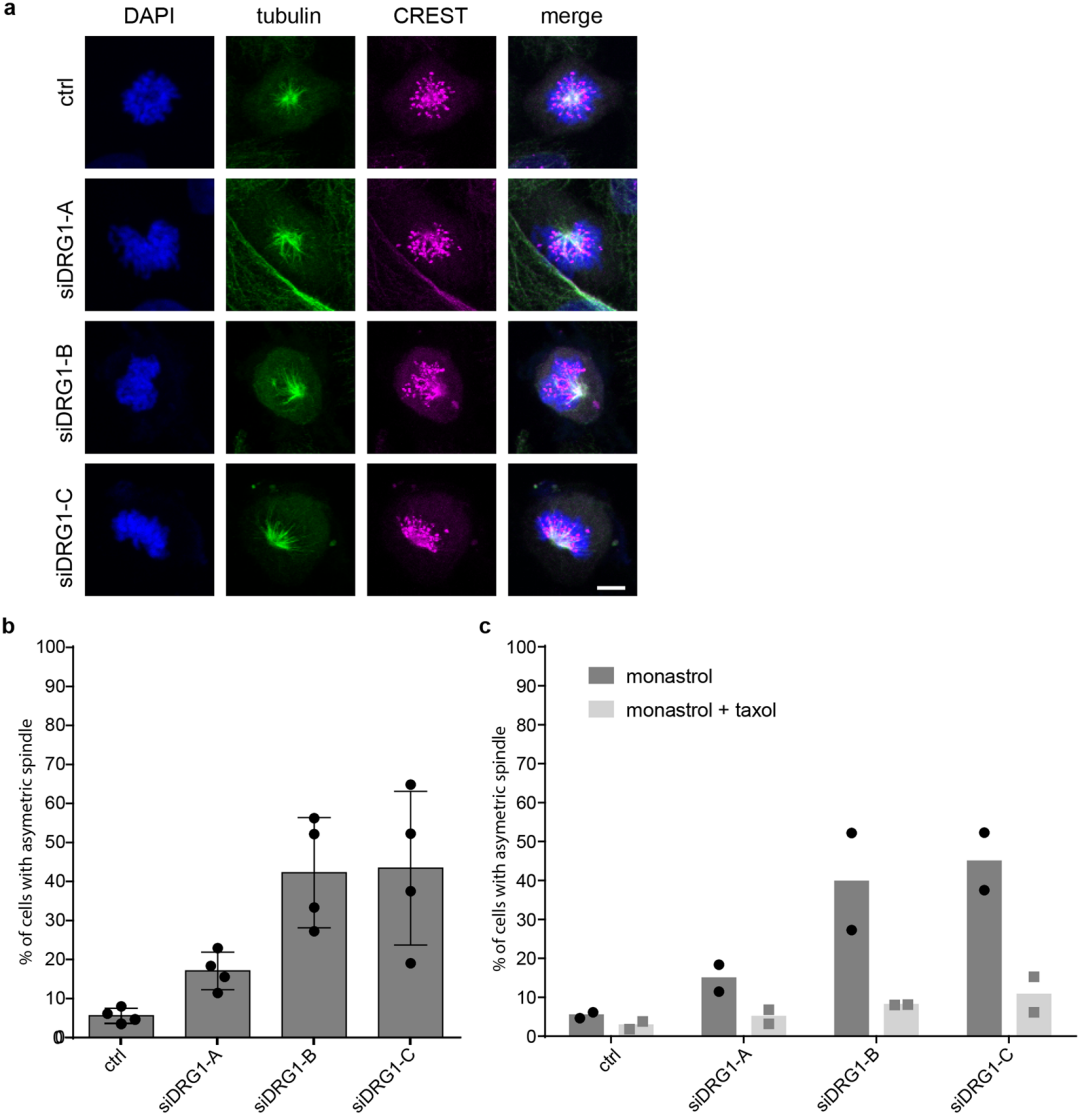
DRG1 plays a role in spindle dynamics *in vivo*. **(a)** HeLa cells were seeded on glass coverslides and transfected with DRG1 or control siRNA oligonucleotides. 72 h post-transfection cells were incubated with 70 µM monastrol with or without 2 nM taxol for 3 h^33^, fixed and stained with antibodies against α-tubulin (green) and anti-human centromere (magenta). DAPI in blue. **(b,c)** Quantitation of cells with asymmetric asters. Z-Stacks from five to eight random positions per condition were acquired and quantified (4 independent experiments for monastrol treatment **(b)** and two independent experiments (represented by the two dots/squares) for monastrol treatment with rescue by taxol **(c)**). Between 15 and 98 cells with monopolar spindles were evaluated per siRNA knockdown per experiment. Mean and SD are plotted. ***P < 0.001; **P < 0.01; *P < 0.01.

Recently, *Stolz et al*.^33^ introduced an assay to identify microtubule plus-end regulators: Inhibition of the mitotic kinesin Eg5 by monastrol, which prevents centrosome separation in the beginning of mitosis, causes monoaster formation. *Stolz et al*. observed that these monoasters are asymmetric if microtubule plus-end assembly rates are increased and that this asymmetry can be rescued by low doses of taxol. We knocked down DRG1 by siRNA and treated the cells with monastrol. Indeed, spindles in cells with reduced DRG1 level showed much more asymmetric monoasters when compared to the control (Fig. 8a and b). This phenotype was also rescued by addition of low doses of taxol (Fig. 8c). This phenotype again suggests an involvement of DRG1 in spindle dynamics.

## Discussion

The function of DRG1 has been long debated. Considering its high evolutionary conservation, it was suggested that DRG1 has an important function in a fundamental cell biological process. We identify here that DRG1 is involved in spindle assembly. DRG1 binds microtubules and can diffuse on the microtubule lattice *in vitro*. DRG1 promotes microtubule polymerization and bundling and stabilizes them. To perform these latter activities, DRG1 does not require GTP hydrolysis, but does require each of its domains as only the full-length protein is functional in these assays. Consistent with these observations DRG1 is also involved in spindle dynamics in HeLa cells: microtubules regrow faster after a cold shock induced disassembly if DRG1 is present; early mitotic progression is extended if DRG1 is downregulated and a high number of asymmetric monoasters forms upon monastrol treatment in cells lacking DRG1.

DRG1 binds directly to microtubules consistent with its previously shown localization at the mitotic spindle^25^. The TGS and HTH domains of DRG1, as well as truncated versions of DRG1 lacking the TGS and/or HTH domain are able to bind microtubules. The S5D2L domain alone is not sufficient to bind to microtubules; however, we cannot be certain that it is properly folded. Electrostatic surface potential analysis shows that DRG1 has a highly positively charged surface stretching over the TGS, the HTH, the S5D2L and the G-domain opposite of the GTP-binding site. Many microtubule binding proteins are highly positively charged. Therefore, this positively charged region might be the binding region of DRG1 to microtubules. This would also explain why most of the domains bind microtubules individually. The interaction of positively charged microtubule binding proteins with microtubules usually occurs via the negatively charged C-terminus of tubulin. However, DRG1 still binds to microtubules lacking the C-terminal ends after subtilisin digestion indicating that this is not the major binding site. Similarly, the *drosophila* non-claret disjunctional (Ncd) kinesin-like protein does not strictly depend on the C-terminus of tubulin for microtubule interaction^34^.

In addition to binding microtubules as an immobile molecule, DRG1 can also diffuse on the microtubule lattice. This behavior resembles e.g. the microtubule depolymerase MCAK^27^. MCAK functions at both ends of microtubules. Its random diffusion towards the ends enhances the chances that MCAK binds microtubule ends compared to simple diffusion in solution. It is possible that DRG1 also targets to the microtubule ends of dynamic microtubule to promote microtubule polymerization there. The proportion of the two different binding modes, immobile and diffusive, depended on the concentration of DRG1. The lower the concentration, the more immobile and the less diffusive DRG1 was. It is unlikely that the two different binding modes represent DRG1 in two different nucleotide bound states, GTP-bound, GDP-bound or nucleotide free, as we observed similar proportions of the two different binding modes in the presence of the non-hydrolysable GTP analogue GTPγS. The two binding modes might perform different functions such as polymerization versus bundling, and could represent different binding sites or different oligomeric states.

While the GTP hydrolysis is not necessary for the microtubule-related functions of DRG1 shown here, the truncated versions of DRG1 have highly reduced or no bundling, polymerization or stabilization activity. It was previously shown that the severe growth phenotype caused by triple deletion of the DRG1 and 2 homologs, Rbg1 and 2 together with the ATPase Slh1 in yeast can be rescued by full-length Rbg1 but not by any of the tested truncations^22^.

The polymerization, bundling and stabilization activities of DRG1 could be completely independent functions or connected to each other: the bundling of microtubules could also stabilize them; the polymerization activity could increase the amount of microtubules in a population that is in the growth phase and thereby stabilize them; the bundling could increase polymerization by increasing the microtubule density close to DRG1. In this respect, it is surprising that the GTPases α- and β-tubulin are directly regulated by another GTPase, DRG1, although not using its GTP hydrolysis activity in this context.

Consistent with the biochemical assays, HeLa cell microtubules that were depolymerized on ice regrew faster if DRG1 was present. This faster recovery can be either explained by the polymerization activity of DRG1 or by the stabilization activity, which might stabilize small microtubule remnants that regrow faster afterwards when cells were provided with fresh, warm medium. It was observed before that DRG1 shows some thermophilic behavior: DRG1 hydrolyzes GTP over a wide range of temperatures with an optimum at 42 °C^23^. Maybe DRG1 is also more active at cold temperatures compared to other proteins or performs its functions mainly under extreme, stress-situations.

Microtubules have important functions in mitosis and interphase. Our *in vitro* data shows that DRG1 has many functions connected to microtubules but the assays cannot distinguish between mitotic and interphasic functions. The cold shock experiment in HeLa cells suggests that DRG1 performs its function in the mitotic spindle, which is confirmed by our observation that the timing from prophase to anaphase is extended in HeLa cells when DRG1 was downregulated by siRNA. Cells treated with monastrol after DRG1 knock down showed a higher proportion of asymmetric spindles compared to the control cells, and this phenotype could be rescued with low doses of taxol. This effect was observed before when negative growth, plus tip regulators were downregulated^33^. In our biochemical analysis, DRG1 promoted microtubule polymerization and acted rather as a positive growth factor. It was shown before for XMAP215 that it can act as a microtubule polymerase or de-polymerase depending on the conditions, like a classical metabolic enzyme catalyzing a reaction theoretically in both direction^35, 36^. We cannot exclude that this is also the case for DRG1. However, as we do not fully understand the reason for asymmetric aster formation, it is more likely that the monastrol assay scores similarly for down-regulating positive and negative regulators.

DRG1 has been suggested to possess a function connected to ribosomes and translation as it co-fractionates with ribosomes. Its function in this context is still not fully understood. The likely independent functions of DRG1 concerning microtubules and translation could be spatially or temporally regulated e.g. DRG1 might have different functions during different cell cycle stages or one of the functions could be induced upon stress situations as previously suggested^16^. Likewise, its function could be regulated by its binding partners.

Together, our analysis shows that DRG1 is a microtubule binding, bundling, polymerization and stabilization factor. It does not need its GTPase activity to perform these functions. Truncated versions bind microtubules but have highly reduced or none of the other activities. Downregulation of DRG1 in HeLa cells indicated that the protein is involved in mitotic spindle assembly. Deregulation of DRG1 was suggested to be involved in cancer formation^25^ and it is conceivable that the function of DRG1 in mitotic spindle assembly is connected to this. It is also possible that the microtubule function of DRG1 is not limited to mitosis. How DRG1 potentially affects interphase microtubule function is an interesting question awaiting detailed investigation.

## Methods

### Protein expression and purification

Constructs for *Xenopus laevis* full-length DFRP1 and DRG1 as well as DRG1 fragments and human full-length DFRP2 were generated from a synthetic DNA optimized for codon usage in *E. coli* (Geneart) and cloned into a pET28a vector or modified pET28a vectors with a SUMO or eGFP-tag. Recombinant protein was expressed in *E. coli* and purified by Ni-affinity chromatography. For fluorescently labeled DRG1, the eGFP-tag was N-terminal. For motility binding assays, the recombinant protein was further purified by ion exchange chromatography (Tricorn High Performance Columns, Mono Q 5/50GL, GE). Proteins were dialyzed against the individual assay buffers.

To gain dominant GTPase mutants, we designed point mutations in the GTPase domain by sequence alignment to other GTPases: to obtain a dominant-negative DRG1 mutant we mutated serine 78 to asparagine, which is a conserved residue that causes a dominant negative mutant e.g. in the small GTPase Ran^37^ and in Rbg1^20, 22^. To obtain a dominant-positive DRG1 mutant we exchanged proline 73 to valine according to the dominant positive mutant of the *Streptomyces coelicolor* GTPase Obg^31^. The DRG1 S78N and P73V mutants were generated by mutagenesis using the QuickChange site-directed mutagenesis kit (Agilent). DRG1 fragments used were aa 49–367 (ΔHTH), aa 1–293 (ΔTGS), aa 1–46 (HTH), aa 293–367 (TGS), aa 175–238 (S5D2L) and aa 49–293 (ΔHTHΔTGS), all based on the *Xenopus laevis* sequence and expressed as His_6_-tagged protein from a pET28a vector.

### Antibodies

Polyclonal antibodies against full-length *Xenopus* and human His_6_-DRG1, *Xenopus* His_6_-DFRP1 and *Xenopus* His_6_-SUMO-DFRP2A were raised in rabbits and used 1:1,000 in western blotting. Antibodies against MEL28/ELYS^38^ and chTOG^39^ as well as CAP-G^40^ have been described previously. The β-actin (A5441), β-tubulin (T7816) and α-tubulin (DM1A) antibodies were obtained from Sigma and the centromere (CREST) antibody (15–234) from Antibodies Incorporated.

### Preparation of taxol-stabilized microtubules

To polymerize microtubules for the co-sedimentation assay, porcine brain tubulin (Cytoskeleton, T240) was resuspended in BRB80 (80 mM PIPES, 1 mM MgCl_2_, 1 mM EGTA, pH 6.8) to 10 mg/ml. The microtubules were polymerized by adding 2 mM GTP and incubation for 30 minutes at 37 °C. Taxol was added to a final concentration of 20 µM. After 10 min incubation, the solution was centrifuged for 10 min at 110,000 x g in a TLA120 rotor (Beckman) and 37 °C. The pellet was resuspended in BRB80 + 20 µM taxol and the concentration was measured using a Bradford assay.

Microtubules for the bundling assay were prepared in a slightly modified way^41^: 10 mg/ml unlabeled tubulin, 2 mg/ml Cy3-labeled tubulin and 1 mM GTP were incubated at 37 °C for 30 minutes. The solution was then diluted tenfold with BRB80 + 20 µM taxol, the microtubules were pelleted by centrifugation for 10 min at maximum speed in a 1.5 ml reaction tube centrifuge at RT and resuspended as above.

### Microtubule binding assay with extracts

HeLa nuclear extracts (4 C Biotech) were diluted with CSF–XB buffer (100 mM KCl, 0.1 mM CaCl_2_, 2 mM MgCl_2_, 50 mM sucrose, 10 mM Hepes, 5 mM EGTA, pH 7.7) to 1 mg/ml. CSF (cytostatic factor arrested)-*Xenopus* egg extracts were diluted 1:3 with CSF-XB buffer. After centrifugation at 100,000 g for 10 min at 20 °C the supernatant was incubated with 2 µM taxol-stabilized microtubules (for CSF extracts 4 µM) at RT for 15 min in the presence of 1 mM GTP and 10 µM taxol. The samples were centrifuged at 100,000 g for 10 min at 20 °C through a cushion of 40% glycerol in CSF-XB containing 20 µM taxol. Pellets were resuspended in wash buffer (CSF-XB buffer containing 1 mM DTT, 1 mM GTP, and 20 µM taxol) and spun for 10 min at 100,000 × g. The washing was repeated one more time. Microtubules binding proteins were eluted with 500 mM NaCl and the pellet and eluate were analyzed by SDS–PAGE and immunoblotting.

### Microtubule binding, bundling and polymerization assays with recombinant protein

The microtubule binding, bundling and polymerization assays were done as in ref. 42. In short, recombinant protein in CSF-XB buffer was incubated with 2 mM GTP (or if indicated GTPγS), with or without 12 µM (or as indicated) taxol-stabilized microtubules and with or without 500 mM NaCl in CSF-XB + 20 µM taxol for 15 min at RT. Afterwards, the solution was spun for 10 min at 100,000 × g in a TLA100 rotor and 20 °C. The supernatant and pellet were analyzed by SDS-PAGE. Coomassie staining of recombinant microtubule binding proteins was quantified using ImageJ.

For microtubule bundling, 0.1–0.3 µM Cy3-labeled microtubules^41^ were incubated with 1 µM recombinant protein in 10 µl BRB80 buffer + 20 µM taxol for 10 minutes at RT. Samples were squashed between a coverslip and slide without fixation and analyzed by confocal microscopy using a LSM780 Zeiss microscope equipped with a Plan-Apochromat 63x/1.4 Oil DIC objective and 561nm-Diode Lasers. For electron microscopy, the samples were stained in 2% uranyl acetate. Images were acquired with a CMOS camera (TemCam-F416, TVIPS, Gauting, Germany) mounted on a Tecnai Spirit (Thermo Fisher Scientific, Eindhoven, The Netherlands) operated at 120 kV.

For microtubule polymerization, 1 µM recombinant protein was incubated with 4 µM porcine brain tubulin and 1 µM Cy3-labeled tubulin and 1 mM GTP for 30 min at 37 °C in BRB80 buffer. The samples were fixed in 400 µl BRB80 buffer containing 0.25% glutaraldehyde, 10% glycerol and 0.1% TritonX-100 for at least 10 min. Samples were spun through 2 ml 25% glycerol in BRB80 for 20 min at 4,600 × g in a Sorvall Heraeus 75002027 K swing rotor and RT onto a coverslip. The coverslips were post-fixed with methanol at −20 °C for 10 min, washed with PBS and mounted with Mowiol. Samples were imaged by a LSM780 Zeiss equipped with a Plan-Apochromat 63x/1.4 Oil DIC objective and 561nm-Diode Lasers.

To digest the taxol-stabilized microtubules with subtilisin, the protease was added to 0.3 mg/ml for 3 hrs. Another 0.3 mg/ml subtilisin were added after the first 90 minutes. The digestion was stopped by addition of 7 mM PMSF and 1:19 of a protease inhibitor mix (10 mg/ml AEBSF, 0.2 mg/ml leupeptin, 0.1 mg/ml pepstatin, 0.2 mg/ml aprotinin) for 15 min. Another 1:12 of protease inhibitor mix was added followed by 40 min of incubation. The microtubules were pelleted by centrifugation, washed several times and resuspended in BRB80 + 20 µM taxol. (Concentrations of taxol-stabilized microtubules were decreased in the co-sedimentation assay to reach for the same amount of subtilisin-digested and not digested microtubules).

### Microtubule motility binding assay

For microtubule polymerization 20–30 μM porcine tubulin were incubated with 5% DMSO, 4 mM MgCl_2_, 1 mM ATP in BRB80 (pH 6.9) for 1 h at 37 °C. Upon finishing, BRB80 supplemented with 10 μM taxol was added to the reaction tube. Afterwards, the microtubules were spun down at 22 psi using a Beckman airfuge. The pellet was re-suspended in BRB80 containing 10 μM taxol.

The flow cell was constructed as described in ref. 43, but the surface was coated with Chlorotrimethylsilane (MTS, Merck Millipore 102333). The flow channels were washed 4–5 times with sterile filtered BRB80 buffer, followed by incubation with anti-β-tubulin (Sigma Aldrich, T7816) for 15–20 minutes at RT. Afterwards, the channels were washed once with BRB80 and blocked using 1% Pluronic F-127 (Sigma-Aldrich, P2443) in BRB80 for 20–25 minutes, followed by 5 times washing with BRB80 and incubation with 10% rhodamine labelled taxol-stabilized microtubules for 15 minutes. The assay buffer (BRB80, 112.5 mM Casein, 1 mM GTP, 20 mM D-Glucose, 250 nM glucose oxidase, 134 nM catalase, 0.5% β-mercaptoethanol) containing the protein was added after a quick wash of the channel. Samples were imaged at 25 °C on a home built total internal reflection fluorescence (TIRF) microscope combined with epifluorescence. The TIRF microscope was equipped with a sCMOS camera (Orca Flash 4.0, Hamamatsu Photonics) and an oil immersion TIRF objective (60x, Nikon). To visualize DRG1 binding, 40 s time-lapse videos were recorded at 10 fps using a continuous image acquisition mode at 100 ms exposure at various concentrations. The fluorophore/protein was excited using 488 laser line (Omicron, LuxX 488-100). Data was primarily processed using Fiji (http://fiji.sc/Fiji). The kymographs were generated by a custom written macro, auto-contrasted, and analyzed for the two different populations. Within our resolution, one fraction appeared to be immobile and one fraction showed diffusive interactions. The criteria for diffusion were that interactions were longer than 0.3 s and appeared qualitatively as “wiggly lines”. The immobile fraction typically also had extended durations and appeared stationary (“vertical lines” in the kymographs). The different populations were manually identified for each concentration from the kymographs. The error bars for the stacked columns for DRG1 proportions were calculated based on a binomial distribution and the error is given by p_i_ (p_i_ − 1)/$\sqrt{N}$, where p_i_ is the probability of one fraction in the whole population and *N* is the total number of events. The residence time was also calculated from the kymographs and box plots were plotted with individual data points overlaid and 5–95 percentile whiskers including the median (horizontal line) and mean (black square). The data was not corrected for bleaching.

### Microtubule polymerization measured by light-scattering

The protocol for the light scattering experiment was adapted from^44^. 1 µM recombinant protein was mixed with 2.5 µM tubulin and 1 mM GTP in polymerization buffer (80 mM PIPES, 2 mM MgCl_2_, 0.5 mM EGTA and 10% glycerol) in a total volume of 200 µl in a 96-well plate with flat bottom. The absorbance at 340 nm and 37 °C was measured for up to 2:15 hrs in a BioTek Synergy H4 Hybrid Multiplate reader. Data was collected every 38 seconds.

### Microtubule stabilization in the cold

The protocol was adapted from^44^: tubulin (12 µM) was polymerized in the absence or presence of recombinant protein (5 µM or as indicated), GTP (1 mM) and DTT (1 mM) in BRB80 buffer for 1 h at 37 °C. Afterwards the sample was incubated on ice for 30 min, followed by centrifugation at 312,000 x g for 20 min at 4 °C. The supernatant and pellet were analyzed by SDS-PAGE. Coomassie staining of tubulin was quantified using ImageJ.

### Cell Culture and transfection

Cell culture experiments were performed according to ref. 45. HeLa cells were maintained in Dulbecco’s modified Eagle’s medium (DMEM) supplemented with 2mM L-glutamine, 10% fetal bovine serum (FBS) and 500 units/ml penicillin-streptomycin (all from Gibco). The H2B–mCherry and tubulin–eGFP cell line^32^ was a gift from Daniel Gerlich (IMBA, Vienna) and was maintained in DMEM supplemented with 2 mM L-glutamine, 10% fetal bovine serum (FBS) and additionally with 0.5 µg/ml puromycin (Gibco) and 500 µg/ml G-418 (Geneticin; Life Technologies). The knockdown experiments were performed with the following siRNA oligonucleotides: siDRG1-1 (HSS107061), 5′-GAAGGCUUUGGCAUUCGCUUGAACA-3′, siDRG1-2 (HSS181476), 5′-CAGCACACCACUUAGGGCUGCUUAA-3′, siDRG1-3 (HSS181477), 5′-CCUGUAACUUGAUCUUGAUUGUUCU-3′ (Thermofisher), and AllStars negative control siRNA (from Qiagen). HeLa cell suspensions were transfected with 40 nM siRNA using Lipofectamine RNAiMAX (Invitrogen) according to the manufacturer’s instructions.

### Live-cell imaging experiments

Live-cell imaging was adapted from^45^. HeLa H2B–mCherry and tubulin–eGFP cells were transfected with siRNA oligonucleotides in 8-well µ-slide chambers (Ibidi). The cells were imaged for 48 h starting at 24 h post-transfection (approx.), using a Plan-Apochromat 20 × NA 0.8 objective and a 488-nm and 561-nm diode lasers on a LSM 5 live confocal microscope (Zeiss) equipped with a heating and CO_2_ incubation system (Ibidi). ZEN software (Zeiss) was used to acquire images from seven 3.6-µm-spaced optical *z*-sections at various positions every 3 min. Then, single position files were generated from the maximum intensity projections in ZEN and converted into image sequences with free licensed AxioVision software (LE64; V4.9.1.0). Segmentation, annotation, classification and tracking of cells progressing through mitosis were performed using the Cecog analyser (http://www.cellcognition.org/software/cecoganalyzer)^32^. The subsequent analysis was performed in Microsoft excel and GraphPad Prism. Three independent experiments were performed.

### Cold shock regrowth experiments

HeLa cells expressing H2B–mCherry and tubulin–eGFP were seeded on glass coverslides and transfected with siRNA oligonucleotides in 24-well well plates (Greiner Bio-One). 72 h post-transfection the cells were incubated on ice for 1 h allowing to depolymerize spindle microtubules^46^. Then, cold media was replaced with warm medium and the cells were incubated at 37 °C. The cells were fixed at indicated times in 4% PFA after one wash with PBS. Afterwards, Z-Stacks (z-scaling 250 nm / Pinhole 26 µm) from ten random prometaphase cells per siRNA, time point and experiment (n = 2) were acquired using a LSM780 Zeiss equipped with a Plan-Apochromat 63x/1.4 Oil DIC objective and 488nm-Argon and 561nm-Diode lasers. The spindle size quantitation in voxels was obtained using Imaris (Bitplane) by absolute intensity based segmentation of the tubulin-eGFP signal in the spindle. The data was exported as excel files and analyzed using GraphPad Prism.

### Evaluation of monoastral mitotic spindles

HeLa cells were seeded on glass coverslides and transfected with siRNA oligonucleotides in 24-well well plates (Greiner Bio-One). 72 h post-transfection the cells were incubated with 70 µM monastrol (Sigma) in the presence or absence of 2 nM taxol for 3 h^33^, washed with PBS and fixed for immunofluorescence with 4% PFA. For immunofluorescence staining samples were incubated for 1 h in blocking buffer (PBS + 0,1% Triton-X100 + 3% BSA). Afterwards the samples were incubated for 2 hrs at RT with anti-α-tubulin (mouse DM1A; Sigma) and anti-human centromere (CREST) (Antibodies Incorporated 15–234) antibodies. As secondary antibodies anti-Alexa-Fluor-488-anti-mouse and anti-Alexa-Fluor-647-anti-human (Life technologies) were used (1 h at RT). After staining with DAPI for 10 min, samples were mounted in mowiol 4–88 (Calbiochem). Z-Stacks (z-scaling 350 nm/Pinhole 25um) from five to eight random positions per siRNA and condition were acquired using a LSM780 Zeiss equipped with a Plan-Apochromat 40x/1.3 Oil DIC M27 objective and 405nm-DPSS, 488nm-Argon and 633nm-Diode lasers. The quantification of asymmetric monopolar spindles is based on at least 4 independent experiments with monastrol treatment and on two independent experiments with monastrol and monastrol + taxol treatment. Per condition between 15 and 98 cells with monopolar spindles were analyzed.

### Statistical analysis for experiments in HeLa cells

When possible the data was tested for normality by D’Agostino & Pearson omnibus normality test and the variances were compared using an F test (P < 0.05). If a Gaussian distribution could be assumed for the data series and they had no significantly different variances, a two-tailed student’s t-test was performed. If a Gaussian distribution could be assumed for the data series and they had significantly different variances, a two-tailed student’s t-test with Welch’s correction was performed. If a Gaussian distribution could not be assumed, a Mann-Whitney test was performed.

## Electronic supplementary material

Supplementary Info
