# Supplementary material for: Developmentally Regulated GTP binding protein 1 (DRG1) controls microtubule dynamics
Source: Sci Rep. 2017 Aug 30;7:9996. doi: 10.1038/s41598-017-10088-5 (PMC5577222; doi:10.1038/s41598-017-10088-5)

Developmentally Regulated GTP binding protein 1 (DRG1) controls microtubule dynamics

Anna Katharina Schellhaus (1, 2), Daniel Moreno-Andrés (1, 2), Mayank Chugh (3), Hideki Yokoyama (1, 2), Athina Moschopoulou (1), Suman De (3), Fulvia Bono (4), Katharina Hipp (4), Erik Schäffer (3), Wolfram Antonin* (1, 2)

*corresponding author

Supplemental material:

Supplementary Figure S1: DRG1 is a microtubule binding protein

(a) 2 µM taxol-stabilized microtubules were incubated with HeLa nuclear extract (NE) and sedimented. The eluates (obtained by 500 mM NaCl) were analyzed by western blotting with the indicated antibodies. **(b)** Coomassie staining of all purified proteins used in this study.

Supplementary Figure S2: Control kymograph without microtubule depicting fluorescent signals

Fluorescent signals that appear as transient bright horizontal stripes (Fig. 2a) occur even in the absence of a microtubule, but in the presence of eGFP-DRG1. This kymograph was generated adjacent to a microtubule in the presence of 4 nM eGFP-DRG1. The frequency of such bright events is more at high concentrations and gradually disappears at low DRG1 concentrations (Fig 2a). The fluorescent signals are indicative of non-specific and transient interactions that take place due to 3D diffusion of DRG1 oligomers. Arrows point to the fluorescent signals.

Supplementary Figure S3: The different microtubule-binding modes of DRG1 exist also in the presence of GTPγS

Motility assays were done as in figure 2 but in the presence of GTPγS. **(a)** Kymographs representing different binding modes (diffusion, immobile) of eGFP-DRG1 over four different concentrations (0.08 nM, 0.4 nM, 4 nM, 40 nM). On top of each kymograph, the respective image of the rhodamine-labelled microtubule is shown. **(b)** The proportions of the different DRG1 binding populations are shown at the aforementioned concentrations. **(c)** Residence time of diffusive and immobile DRG1 molecules on microtubule lattice are 9.3 ± 0.8 s (mean ± S.E.M., 0.08 nM), 8.4 ± 0.8 s (0.4 nM), 9.4 ± 0.8 s (4 nM), 5.9 ± 0.6 s (40 nM) for the immobile fraction and 2.4 ± 0.3 s (40 nM) for the diffusive population. Color scheme: diffusion (cyan), immobile (green). Exemplary events are pointed out by arrows.

Supplementary Figure S4: DRG1 interacts with microtubules mimicking the GTP state

(a) Rhodamine labelled GTPγS microtubules (left) and 4 nM eGFP-DRG1 (right) qualitatively show no enrichment for microtubules mimicking a GTP cap state as compared to **(b)** rhodamine labelled GTP-

taxol stabilised microtubules (left) at same concentration of eGFP-DRG1. **(c)** and **(d)** show exemplary kymographs from the data represented in **(a)** and **(b)** for the GTPγS and GTP state, respectively.

Supplementary Figure S5: MT stabilization depends on the DRG1 concentration

12 µM tubulin were polymerized in the presence of indicated amounts of DRG1 for 1 h at 37 °C and placed on ice for 30 min. MTs were then pelleted by centrifugation, while free tubulin stayed in the supernatant. Pellet and supernatant were analyzed by SDS-PAGE. The quantification shows the tubulin fraction found in the pellet. Columns represent the average of three independent experiments with the individual data points indicated.

Supplementary Figure S6: The GTPase activity of DRG1 is not necessary for its microtubule associated functions

(a) MT co-sedimentation was done as in figure 1b with DRG1 S78N and DRG1 P73V. The quantification shows the fraction of the proteins in the pellet, DRG1 WT data are from the same experiments. Columns represent the average of three independent experiments, individual data points are indicated. **(b)** MT co-sedimentation was done as in figure 1b with DRG1 WT in the presence of GTP or GTPγS. The quantification shows the fraction of the proteins in the pellet. Columns represent the average of four independent experiments, individual data points are indicated. **(c)** Light-scattering experiment was performed as in figure 5d with DRG1 WT and DRG1 S78N.

Supplementary Figure S7: Full-length DRG1 is necessary to bundle, polymerize and stabilize MTs

Truncated DRG1 versions were tested in the MT bundling **(a)**, polymerization **(b)** and stabilization **(c)** assays. The quantification in **(c)** shows the tubulin fraction in the pellet. Columns represent the average of three independent experiments, individual data points are indicated.

Supplementary Figure S1

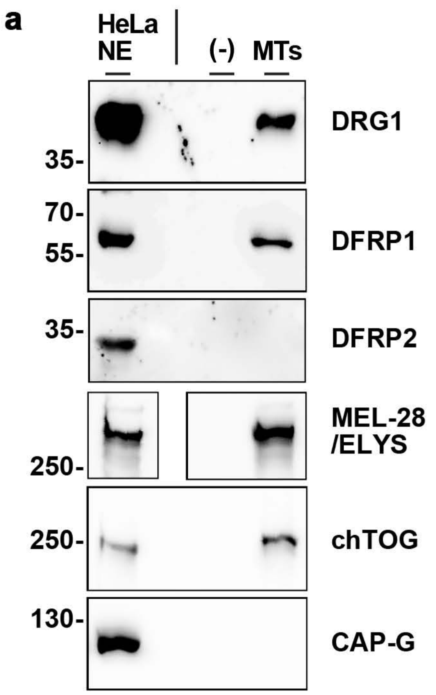

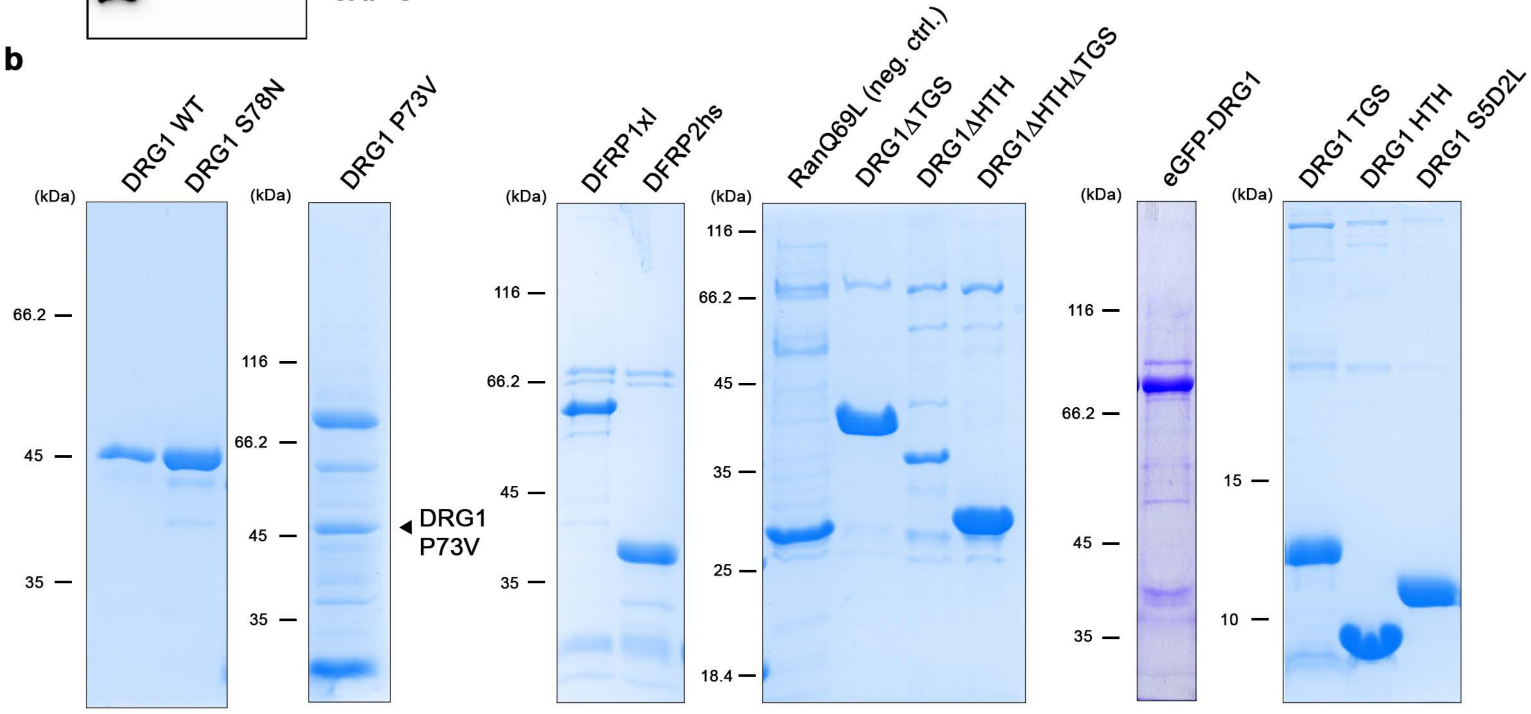

Supplementary Figure S2

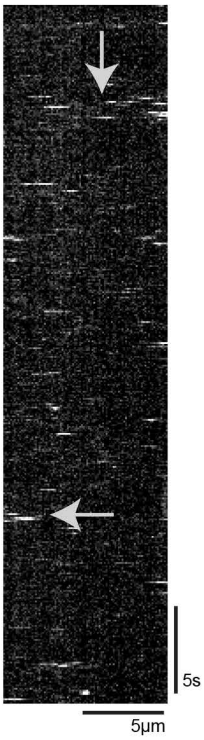

Supplementary Figure S3

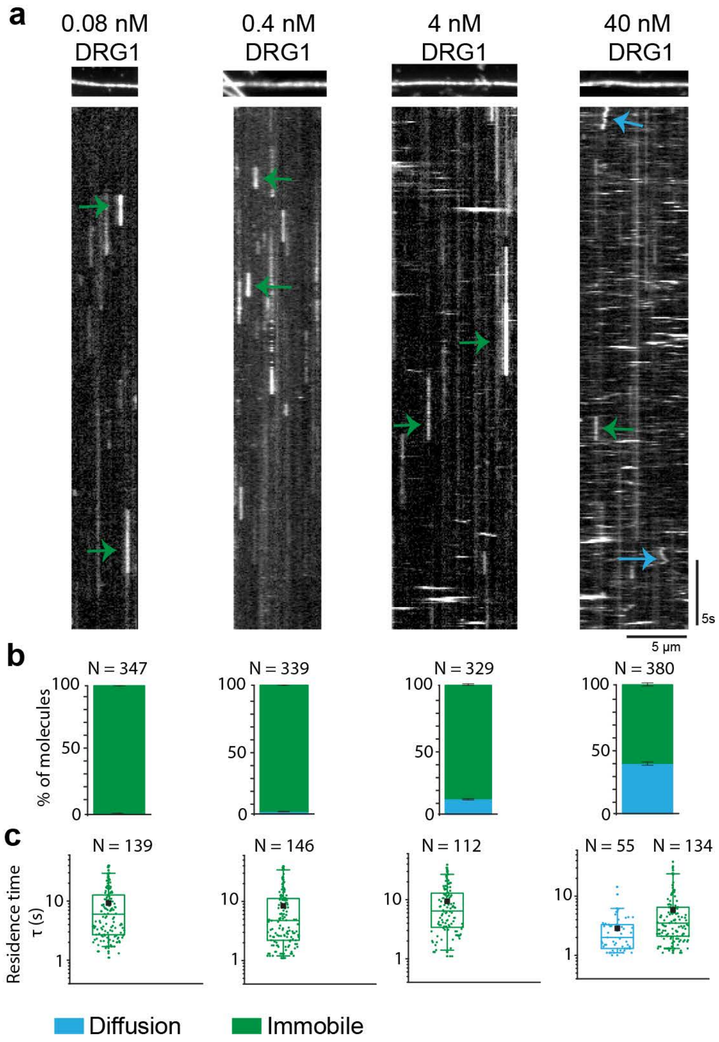

Supplementary Figure S4

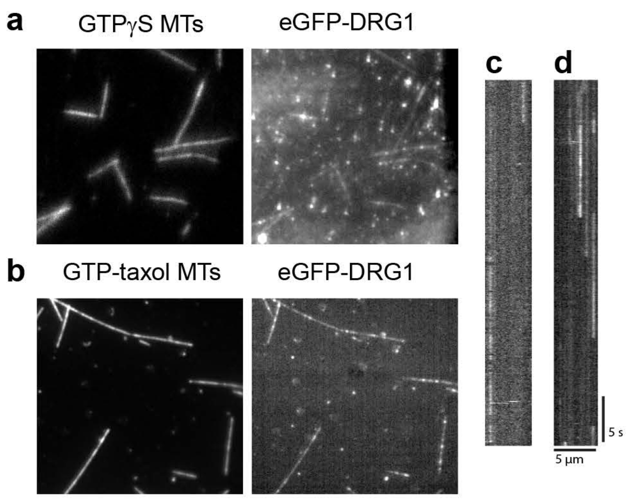

Supplementary Figure S5

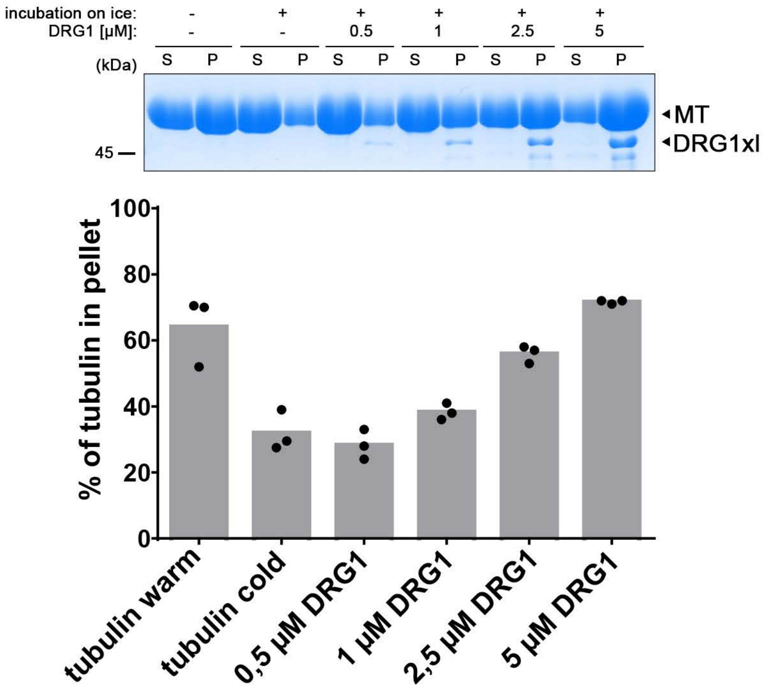

Supplementary Figure S6

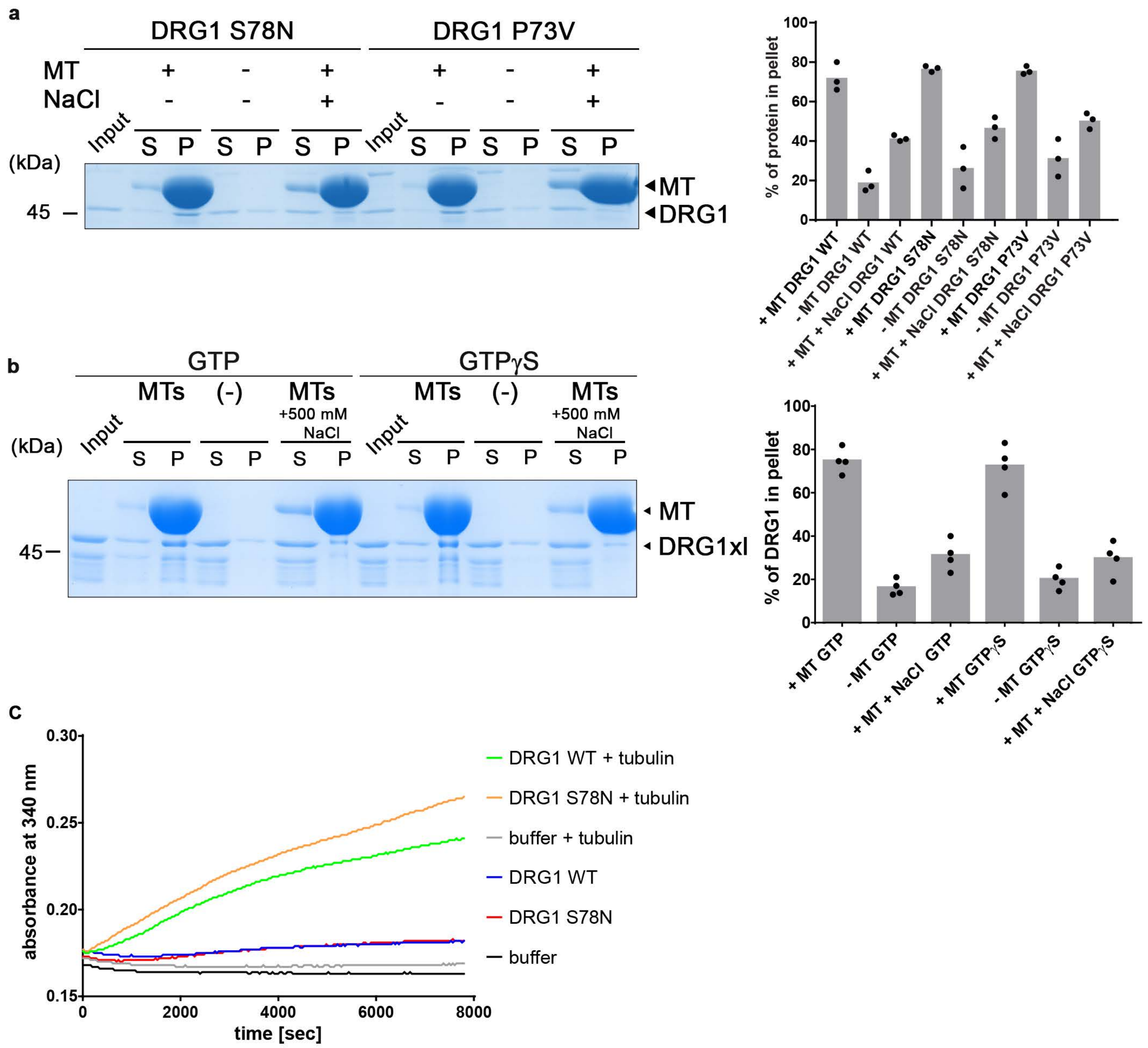

Supplementary Figure S7

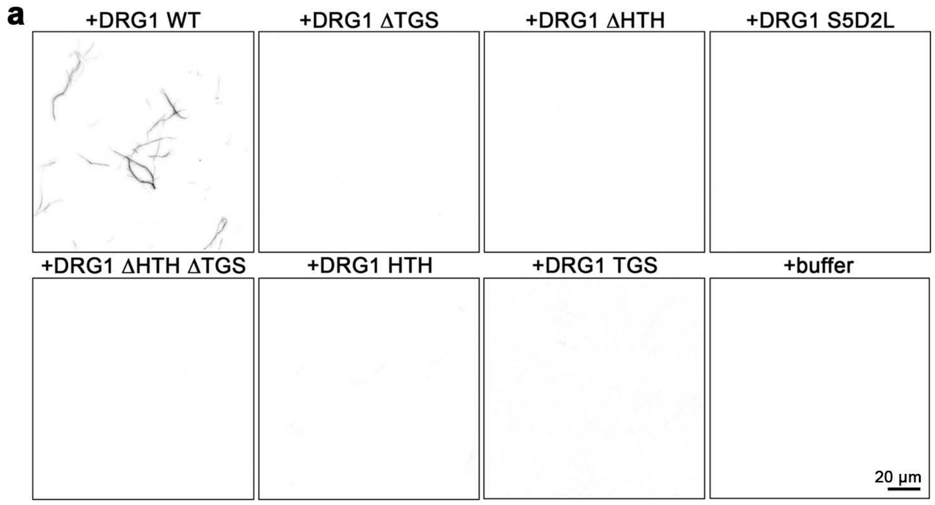

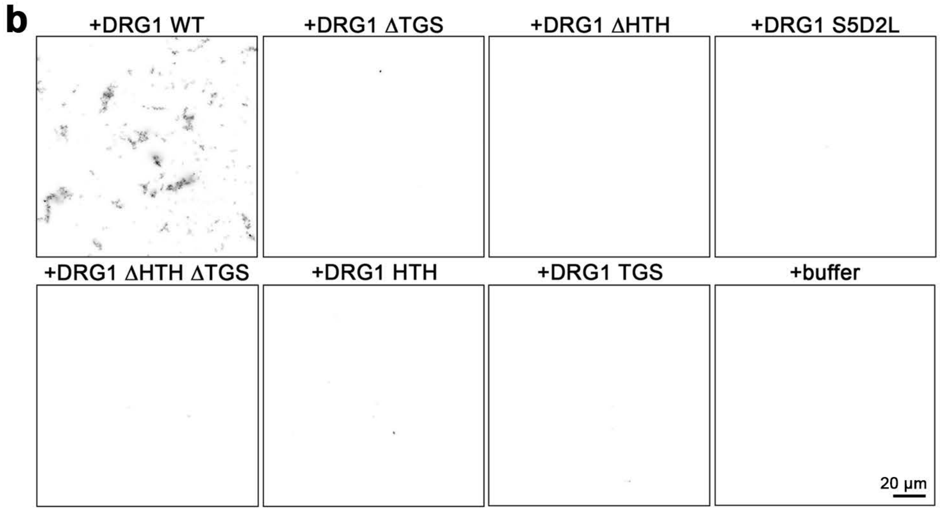

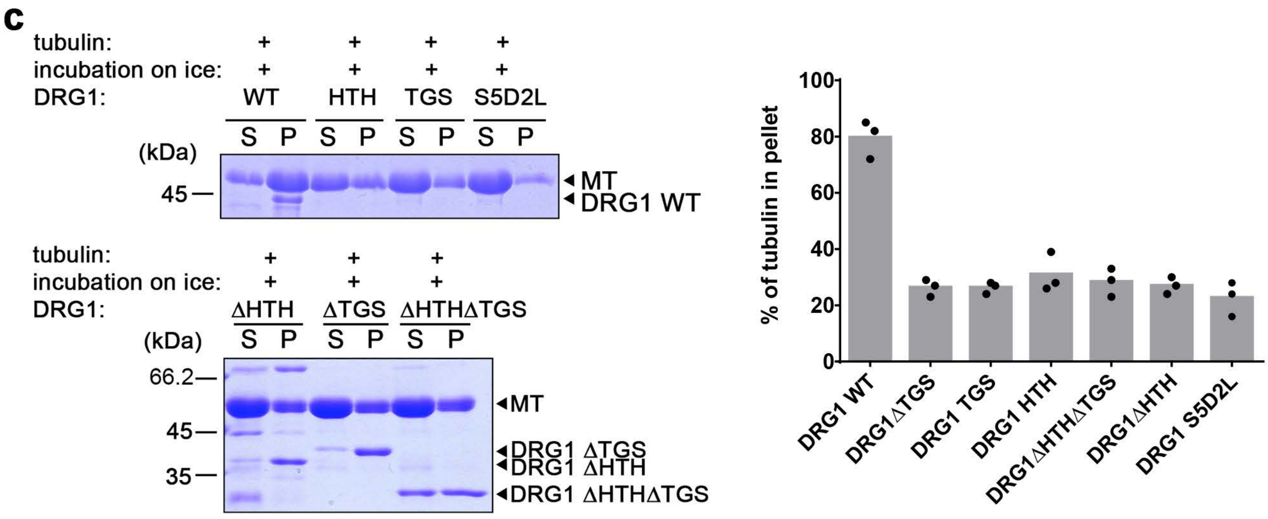

Supplement: Supplementary file 1 — Supplementary Info [file 41598_2017_10088_MOESM1_ESM.pdf]
